# Automatic Localization of Soybean Seedlings Based on Crop Signaling and Multi-View Imaging

**DOI:** 10.3390/s24103066

**Published:** 2024-05-11

**Authors:** Bo Jiang, He-Yi Zhang, Wen-Hao Su

**Affiliations:** College of Engineering, China Agricultural University, Haidian, Beijing 100083, China; jb6011978@gmail.com (B.J.); zhangheyi@cau.edu.cn (H.-Y.Z.)

**Keywords:** computer vision, system crop signal, rapid plant detection, precision agriculture

## Abstract

Soybean is grown worldwide for its high protein and oil content. Weeds compete fiercely for resources, which affects soybean yields. Because of the progressive enhancement of weed resistance to herbicides and the quickly increasing cost of manual weeding, mechanical weed control is becoming the preferred method of weed control. Mechanical weed control finds it difficult to remove intra-row weeds due to the lack of rapid and precise weed/soybean detection and location technology. Rhodamine B (Rh-B) is a systemic crop compound that can be absorbed by soybeans which fluoresces under a specific excitation light. The purpose of this study is to combine systemic crop compounds and computer vision technology for the identification and localization of soybeans in the field. The fluorescence distribution properties of systemic crop compounds in soybeans and their effects on plant growth were explored. The fluorescence was mainly concentrated in soybean cotyledons treated with Rh-B. After a comparison of soybean seedlings treated with nine groups of rhodamine B solutions at different concentrations ranging from 0 to 1440 ppm, the soybeans treated with 180 ppm Rh-B for 24 h received the recommended dosage, resulting in significant fluorescence that did not affect crop growth. Increasing the Rh-B solutions reduced crop biomass, while prolonged treatment times reduced seed germination. The fluorescence produced lasted for 20 days, ensuring a stable signal in the early stages of growth. Additionally, a precise inter-row soybean plant location system based on a fluorescence imaging system with a 96.7% identification accuracy, determined on 300 datasets, was proposed. This article further confirms the potential of crop signaling technology to assist machines in achieving crop identification and localization in the field.

## 1. Introduction

Soybean is the fourth most important grain crop grown across the world [1]. As one of the most profitable agricultural products, soybean plays an important role in serving as a primary source of protein and nutraceuticals for both humans and livestock, and is produced widely around the world [2,3]. Weeds that grow spontaneously on agricultural soils compete fiercely with soybeans for light, water, and nutrients in the early stages of soybean growth, which greatly inhibits the growth of soybeans [4]. The most severe impact of weeds on soybean growth occurs within three weeks after soybean planting, causing adverse effects on soybean yield and quality [5]. Since humans depend directly or indirectly on soybean crops for food production, severe losses in soybean yield may impact global food security. Considering the importance of soybeans in agriculture, their productivity and product quality must be maximized. Therefore, early weeding operations in soybean fields are very important for soybean growth and yields.

The existing methods of weeding include manual, chemical, and mechanical weed control. As the most traditional method, manual weeding can be effective in small fields [6]. Because of its high labor intensity and low labor efficiency, manual weeding is slow or insufficient to meet the needs of large-scale modern agriculture [7]. Moreover, the scarcity of the agricultural labor force is resulting in the increasing labor cost of manual weed control, making it less attractive [8]. Currently, there is a growing reliance on uniform applications of chemical herbicides for weed control [9]. This is a source of concern because a substantial amount of the herbicides are dispersed in the air and soil, causing serious harm to the environment and other organisms [10]. Additionally, the application of herbicides is contributing to the growing issue of weed resistance, posing a significant risk to ecological safety [11]. Mechanical weeding is environmentally friendly but the lack of rapid and precise crop positioning technology results in our inability to accurately remove intra-row weeds, potentially causing unintended damage to the crops themselves [12,13]. Therefore, a rapid and precise plant detection and location technique is imperative for mechanical weeding devices. Fast and accurate crop positioning technology is crucial for automated precision weeding equipment, which is an indispensable part of modern agriculture.

With the advancement of image acquisition technology and image processing technology, computer vision technology has made significant progress in the field of weed detection [14,15]. For instance, He et al. identified weeds with 96% accuracy, using features such as the shape, texture, and fractal dimension of weeds via a multi-feature fusion method [16]. Zhang et al. introduced an innovative approach to segment and extract weeds amidst complex backgrounds, utilizing R-B (Red and Blue) color difference features as the foundation of their method [17]. Zhang et al. proposed an automated weed control system using hyperspectral species identification [18]. In the outdoor test, 2.4% of plants were significantly damaged by a speed of 0.04 m/s, which is very slow. However, the traditional methods of computer vision are limited by the differences in the profile or color of crops and weeds; it would drop to a level of poor identification accuracy when the crop is similar or adjacent to weeds. In recent years, with the development of computer calculation capabilities, deep learning techniques have become more favored. A significant number of crop field weed detection models incorporating deep learning and computer vision techniques have been proposed, with positive results, in recent years [19,20,21,22,23,24,25]. In recent years, with the development of big data technology and the improvement of computers’ computing performance, as well as breakthroughs in deep learning algorithms, researchers both domestically and internationally have conducted extensive research on the identification and localization of plants using computer vision and deep learning techniques. For example, Alessandro dos Santos Ferreira et al. created a dataset which contains over fifteen thousand images of the soil, soybeans, and broadleaf and grass weeds. they used CaffeNet architecture to train the Neural Network AlexNet, and this work achieved above a 98% accuracy using ConvNets in the detection of broadleaf and grass weeds in soil and soybean [26]. Zhang et al. employed the improved SE-YOLO5x algorithm for the identification and localization of vegetables and weeds, achieving an accuracy rate of 97.14% [22]. Wang et al. combined YOLO v5 with an attention mechanism in a deep learning approach to the real-time detection of invasive weed *Solanum viarum* seedlings in the field, achieving a detection accuracy of 94.65% [27]. Fang et al. collected 4694 representative images of cotton field scenarios and instructed a novel deep learning model that combined a CBAM (convolutional block attention module) module, a BiFPN structure, and a Bilinear interpolation algorithm. The proposed network can effectively learn deep information and distinguish weeds from cotton seedlings in various complicated growth states and reach an mAP (mean average precision) of 98.3% on the dataset they constructed [28]. Reenul Reedha et al. utilized a self-attention mechanism and transfer learning algorithms for weed and crop image recognition, but they found that transfer learning algorithms performed well only when applied to large datasets [29]. Although deep learning techniques exhibit significant potential in weed detection, achieving high accuracy in weed identification requires a substantial volume of pre-training data. Hasan et al. surveyed 70 relevant research works and found that most studies utilized supervised learning with advanced deep learning models, achieving improved performance and classification accuracy through fine-tuning pre-trained models on plant datasets. However, the research a achieved high accuracy only in limited experimental setups, such as small datasets of specific crops and weed species [30]. Aside from the collection, annotation, and maintenance of datasets entailing significant manpower and time costs, the accuracy of the model is highly dependent not only on the algorithm itself but also on the quality of the dataset and its annotations. Moreover, the current recognition models are mostly specific to identifying weeds in particular crops, resulting in their poor generalization ability. In addition, deep learning models are typically presented as black boxes with poor interpretability; the recognition performance of deep learning models heavily relies on the computational power of their hardware [30]. In complex environments such as uneven terrains and occlusions, it is difficult to ensure their high accuracy. The research methods mentioned previously possess inherent strengths and weaknesses. Nevertheless, a common requirement across all approaches is the need for a substantial dataset for pre-training. Furthermore, it is crucial to note that the generalizability of the model cannot be assured.

Crop signaling is a novel technology aiming to create a unique signal for plants that can be captured by specific sensors [31]. Unlike image processing and analysis using features such as color and texture, crop signal technology is a novel approach that utilizes dyes or fluorescent compounds with different colors or fluorescence from the crops themselves to create machine-readable signals [32]. Crops marked with these crop signals can generate a fluorescence distinct from weeds under specific excitation light sources. By capturing these signals using specific sensors, the field localization of crops can be achieved. This technology was first proposed by Nguyen et al. in 2017 as a new solution for automatic weed control in vegetable crops [33]. In their research, the crop plants marked during field planting produced unique optical signals, which successfully facilitated the differentiation between crops and weeds.

Fluorescent labeling mainly includes physical labeling, biological labeling, and chemical labeling [34]. Physical labeling refers to fluorescent straws fixed next to seedlings or topical markers sprayed on the stems and leaves of lettuce and tomato plants [35,36,37]. Computer vision algorithms based on top-view and six side-view images were developed to identify plant stems, indirectly achieving plant localization. The crop signal system was able to recognize 97.8% of crops at a speed of 3.2 km/h [34]. Labeling each lettuce or tomato plant is time-consuming; it is also hard to avoid labels being removed due to irrigation or adverse weather conditions such as wind and rain. Biological labeling refers to introducing exogenous genes capable of producing fluorescent proteins into crop cells in order to express them as markers [35]. However, this method involves genetically modified crops, which are not well understood in terms of environmental and food safety issues. Based on this, Su et al. updated the technology using fluorescent compounds, which involves marking crops with plant-friendly fluorescent molecules that can be absorbed by the seeds or seedling roots and transported systemically within the plants [36,37]. These crop markers generate a special fluorescence distinct from chlorophyll fluorescence under the same excitation light. Rhodamine B (molecular weight: 479, log K_ow_ = 1.5, λ_ex_/λ_em_ = 555/582 nm, abbreviation: Rh-B), a chemical marker with unique optical properties, was used by Su et al. to label crop plants, successfully establishing a recognition technique for celery [36,37]. However, for direct-seeded crops, root treatment techniques cannot be used. A previous study found that the seed coat of beans and soybeans has a strong permeability to Rh-B, which can be absorbed by these seeds [38]. Su et al. applied Rh-B as a tracer to beans and successfully generated machine-readable signals in the cotyledons and main stems of bean seedlings [39]. However, these signals are easily obscured by growing leaves, making them not visible from a top-view perspective.

In summary, machine vision technology has great potential in soybean weed detection. However, traditional machine vision technology is limited to color, texture, and shape characteristics and cannot accurately classify weeds with similar shapes or overlapping weeds. Crop signaling technology provides crops with unique signal markers that are different from those of weeds, making the identification and classification of crops and weeds more accurate and faster. Existing crop signaling technologies mostly focus on surface spraying or crop labeling methods, which are easily affected by the environment and fall off, causing the crops to lose their unique labels, making it impossible to identify crops and weeds. Therefore, this study further studies the basis of crop signals and proposes a new systematic crop signal technology. It is a labeling technology that uses fluorescent signal molecules to treat crop seed seedlings so that they can remain present in the crop.

This study further develops soybean detection and localization techniques based on systemic crop signaling technology. The key innovations of this study are (1) the performance of a systematic analysis of the impact of Rh-B on soybean growth, (2) the examination of Rh-B as a signaling compound and its fluorescent distribution patterns during the early stages of soybean seedling growth, and (3) the proposal of a soybean detection algorithm based on computer vision and a multi-view three-dimensional soybean localization algorithm. To the best of the authors’ knowledge, this use of systematic crop signaling combined with a multi-view algorithm was the first technique used to separate soybeans from weeds. 

## 2. Materials and Methods

### 2.1. Seed Treatment and Seedling Culture

The soybean seeds (Variety: ZH13, provided by Fenghong Seed Industry, Weifang, Shandong, China) used in the experiments were preselected based on the criteria of a uniform size and full grains. The soybean seeds were first treated with the fungicide Captan (Xisian, ADAMA, Ɪzreɪl) to clean out germs three times. In each group of experiments, the soybean seeds were divided into six groups after being rinsed three times using deionized (DI) water. Soybean seeds were immersed in various concentrations of Rh-B dye for different durations to assess how different seed treatment methods affected the growth of soybean seedlings and their fluorescence properties. The control group was treated with DI water. The Rh-B solution used in the experiments was prepared just before the seeds were soaked. All the soybeans were placed in a ventilated area for 3 h after soaking to filter out excess moisture. For the first experiment, different densities of the Rh-B solution, 0, 60, 90, 120, 150, and 180 ppm (ppm = mg·L^−1^), were selected, with a soaking time of 24 h. Then, the 90, 180, 360, 720, and 1440 ppm densities of the Rh-B solution were selected with an immersion time of 24 h in the second experiment. Based on the above two experiments, a suitable solution of Rh-B was chosen to soak soybean seeds for different times between 12 and 60 h. To control the variables, the long soaking groups were prior-treated.

The treated seeds were germinated in six seedling boxes grouped by the soaking time/density of Rh-B. The soybean seeds were placed on a sieve board covered with filter paper. A total of 5 mL of DI water was dropped into the seedling box to initiate germination. The lid of the seedling box was covered, and it was placed in the dark at a temperature of 23 ± 1 °C. The soaked seeds were placed in a seedling tray to germinate and their germination observed three days later to evaluate the seed germination rate. After 3 days, the germinated soybean seeds were removed and planted in a nursery tray filled with moist nutrient soil under a plant grow light quantum board (Xueqiu, 450 W, Zhongshan, Guangdong, China). The temperature of the laboratory was 25 °C with a humidity of 56%rh. In the second and third experiments, the seeds were planted in moist nutrient soil directly after being treated with Rh-B, which was grown outdoors under sunlight in Haidian District, Beijing. The soybeans soaked in different concentrations of Rh-B were sown in the same soil, all soybeans were kept at a consistent daily watering rate, and their biomass was measured after 30 days of incubation. All the experiments were repeated three times.

### 2.2. Acquisition of Fluorescence Images 

**Figure 1:**
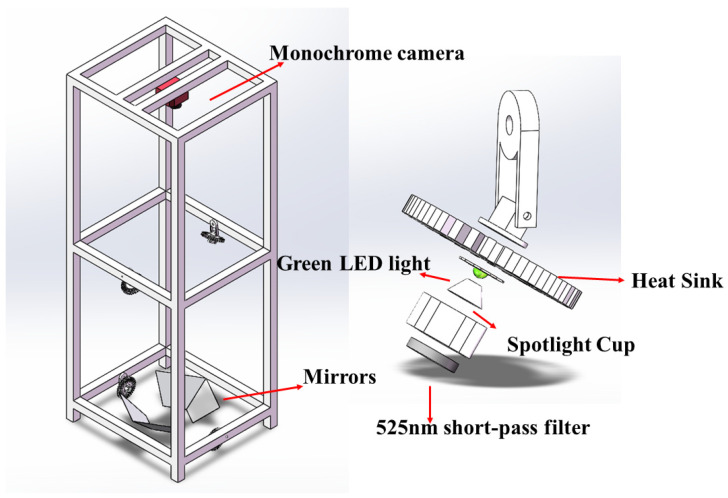
Schematic diagram of the framework of the fluorescence imaging system.

The capture of fluorescence images was meticulously performed within a purposefully designed imaging system. Figure 1 shows the framework of the identification device. The designed imaging chamber consists of a dark box (about 0.6 m by 0.6 m by 1.8 m high), four green (523 nm) light-emitting diode (LED) lights (Model LZ4-40G108-0000, LED Engin Inc., San Jose, CA, USA), and a cooled monochrome complementary metal–oxide semiconductor (CMOS) camera (ASI1600GT, ZWO Inc., Suzhou, China). The four green LEDs were positioned at four locations in the system, at an appropriate altitude and illumination angle. A short-pass filter (550 nm) was set in front of each LED to filter out light exceeding 550 nm. The four LEDs, powered by a stable AC to DC power supply (12 V, Schneider Electric, Suzhou, China), use a constant current module to guarantee equal light intensity. The fluorescence images were captured by a CMOS camera set in the middle of the top of the dark box. The camera utilized for the study has a Panasonic MN34230 sensor (ZWO Inc., Suzhou, China) with a resolution of 4656 × 3520 pixels and a pixel size of 3.8 µm, equipped with a lens (Nikkor 50 mm f/1.8 D, Nikon, Tokyo, Japan). In addition, the camera featured an internal 5-position ultra-quiet electronic filter wheel and a 12 V power distribution capability. To mitigate unwanted ultraviolet (UV) irradiation, a long-pass UV filter (Model Zeta L41, Kenko Co., Ltd., Tokyo, Japan) was affixed to the front of the camera lens. This filter effectively absorbed irradiation up to 410 nm. To maintain low noise levels, the camera employed a regulated two-stage thermoelectric cooler (TEC) cooling system, enabling the sensor’s temperature to be lowered to 40 °C below ambient temperature. A 31 mm, single-band bandpass filter with a center wavelength of 575 nm was placed in the camera’s filter wheel to allow Rh-B fluorescence to pass through. The filter wheel was operated by a high-grade stepper motor, known for its whisper-quiet performance. The imaging system can be controlled outside of the dark box by a computer software called Astronomy Common Object Model (ASCOM 1.10.1). The four LEDs can be controlled by a switch outside of the dark box. The camera focal length and light source height are adjusted in advance to obtain the best imaging effect. 

### 2.3. Image Pre-Processing and Pseudo-Color Image Generation

The obtained grayscale images of soybeans were pre-processed using Fiji software 1.53t (Fiji ImageJ, National Institutes of Health, Bethesda, MD, USA). To ensure image accuracy, a black cloth was positioned at the base of the dark box as a background. A smoothing operation was initially applied to remove noise. Next, the region of interest (ROI) was delineated from the background through the application of a global threshold. Subsequently, the “analyze” and “measure” functions were employed to obtain fluorescence intensity metrics, including the mean, minimum, and maximum values. Following the acquisition of these results, the Image- > Lookup Tables- > ICA option was utilized to generate a pseudo-color representation of the image.

### 2.4. Improved Multi-View Positioning Algorithm

A rapid and precise multi-view plant center location algorithm based on systematic plant signaling technology was proposed for detecting and locating the soybean seedlings. To simulate field conditions, soybean plant images were captured at various positions within the imaging system. Each image of soybeans was captured by a monochrome camera with an exposure time of 50 ms under the illumination of a four-point green excitation light source. Moreover, each image was divided into 6 main areas with information about the position of the mirror edges. The six areas were the top left mirror area (LT), bottom left mirror area (LB), top right mirror area (RT), bottom right mirror area (RB), central crop area (MC), and background area (BG). In the context of the six designated regions, LT and RB, as well as LB and RT, are considered pairs of mutually opposing mirror regions. The unique fluorescence of the systematic crop signaling technique, while not detected in the background region, will be observed and detected in the five other regions. The simultaneous observation of five views will greatly prevent the crop signal fluorescence from being obscured by a high density of weeds.

**Figure 2:**
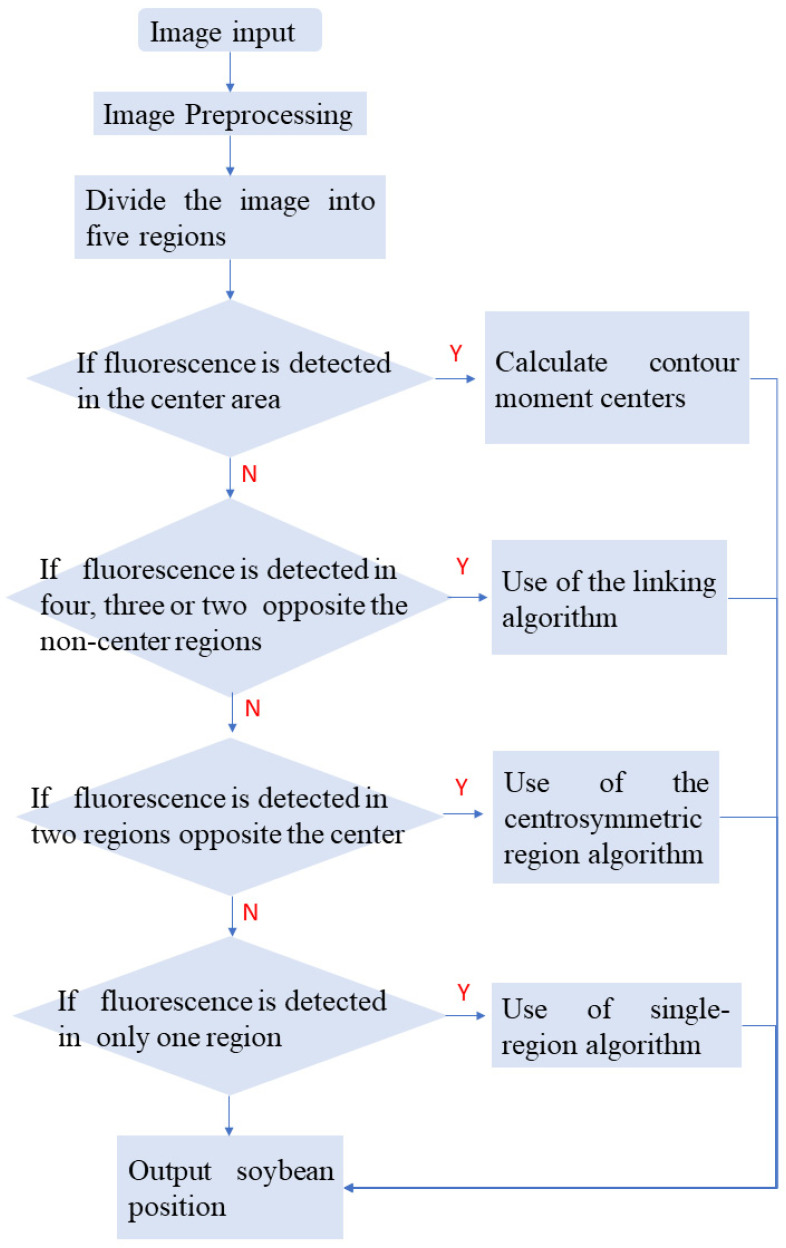
The flow chart of the soybean seedling detection algorithm.

This threshold segmentation technique was applied to extract the center of the fluorescence signal in each region before removing the image noise via smoothing and filtering operations. The camera used in this experiment could capture grayscale images with a single channel bit depth of 8. With a bandpass filter installed in front of the lens, the camera captured only the image information located in a wavelength range of plus or minus 25 nm from 575 nm. Therefore, most of the image intensity, except for the excitation fluorescence, was much lower than the image intensity of the crop signal region. The crop signal location information could be extracted quickly and accurately using the threshold segmentation operation. After extracting the fluorescence position information of multiple regions of interest, the algorithm shown in Figure 2 was used to accurately obtain the position information of the soybean plant. In the process of identifying and locating soybean plants, seven main scenarios occurred: (a) fluorescent information in the middle region, (b) no fluorescent information in the middle region but fluorescent information in four different mirror regions, (c) no fluorescent information in the middle region but fluorescent information in three different mirror regions, (d) no fluorescent information in the middle region but fluorescent information in the two mirror regions facing each other, (e) no fluorescent information in the middle region but fluorescent information in the two mirror regions that were not opposite each other, (f) no fluorescent information in the middle region but fluorescent information in a single mirror region, (g) and no fluorescent information at all. When there was fluorescence information in the central region, the position of the fluorescence in the central area was extracted directly as the crop position. In scenario b, the procedure involved connecting the centers of the fluorescence ROIs within each opposing pair. In scenario c, apart from connecting the centers of the fluorescence ROIs in opposing pairs, an additional step entailed drawing a vertical line from another fluorescence ROI center to the mirror’s edge. In scenario d, a vertical line was drawn for each individual center of the fluorescence ROIs. In these three scenarios, the point of the intersection of the line segments was regarded as the precise location of the crop.

**Figure 3:**
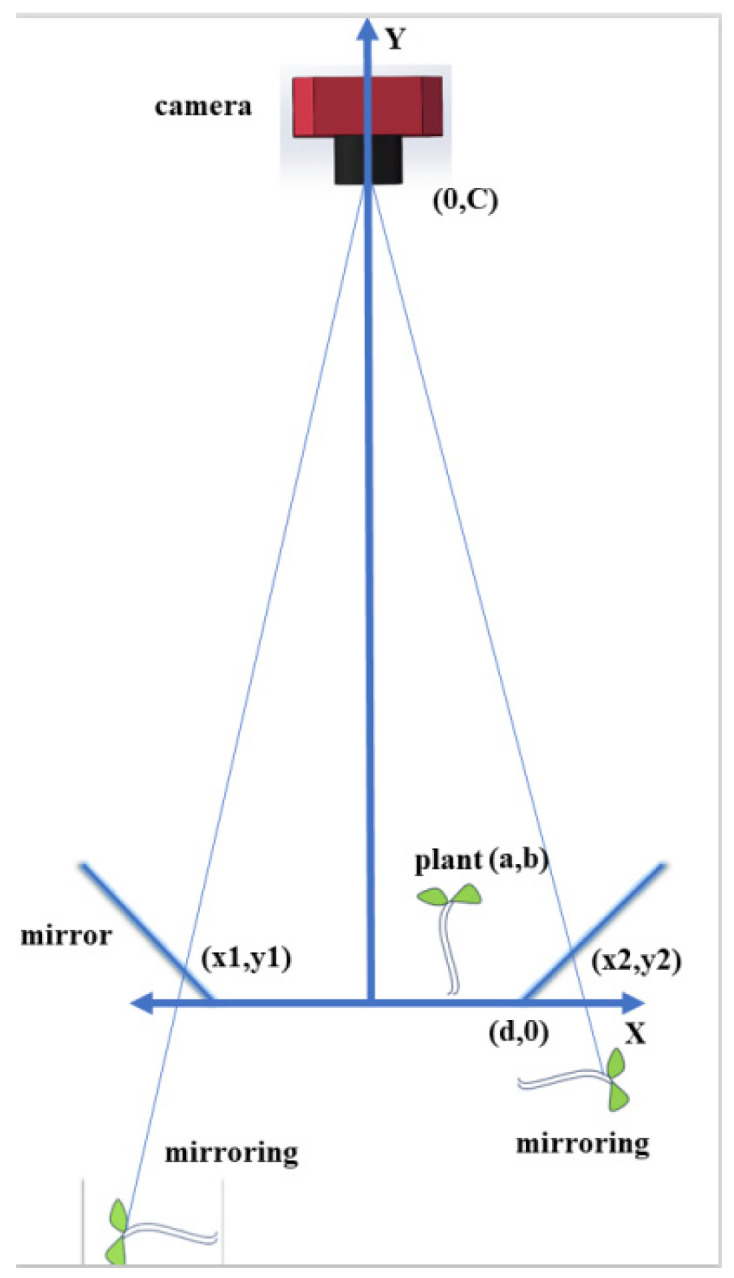
Schematic diagram of mirror reflections from oppositely placed mirrors.

However, when fluorescence was detected in only two mirrors positioned opposite each other or in a single mirror, it became infeasible to determine the plant’s centroid using the standard connecting-line approach. As a result, two distinct algorithms were introduced to calculate the plant’s position based on the coordinate differences of the fluorescent positions within the mirrors. Specifically, the coordinate differences of the fluorescent positions in the two-mirror scenario were captured in a single frame, whereas in the one-mirror scenario, the coordinate differences were captured across two frames with a certain interval. Figure 3 shows the principle of solving coordinate differences in fluorescence in opposing mirrors.

In the opposite mirror cases, the position of the actual coordinate points of the crop in relation to its fluorescence obeys the following equations:

(1) y=x−d,

(2) $$\frac{x}{b}+d=\frac{y-c}{a-d-c},$$

(3) $$x=\frac{bc+dc+bd+d^{2}}{-a+b+c+2d},$$

(4) $$x1=\frac{bc+dc+bd+d^{2}}{b-a-c},$$

where *c* represents the camera height; a represents the actual position of the fluorescence; *b* represents the height of the fluorescence relative to the reference plane; *x* and *x*1 represent the difference of fluorescence in the two mirrors, respectively; and *d* represents the distance from the bottom of the mirror to the center in the reference plane.

**Figure 4:**
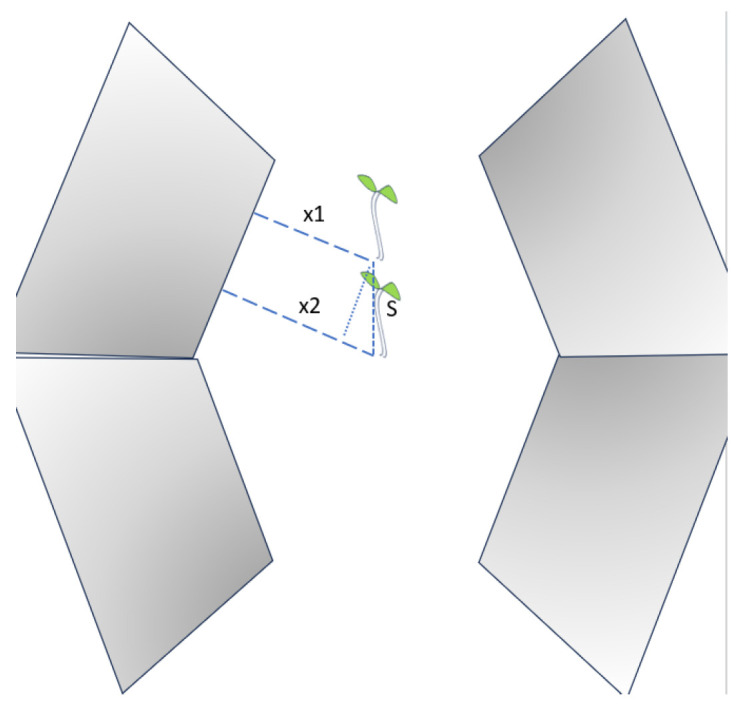
Schematic diagram of coordinate differences across different frames of fluorescence in a single mirror.

The principle of one-sided mirror recognition is shown in Figure 4. In the one-mirror cases, the position of the actual coordinate points of the crop in relation to its fluorescence obeys the following equations:

(5) $$x1=\frac{bc+dc+bd+d^{2}}{-a+b+c+2d},$$

(6) $$x_{2}=\frac{bc+dc+bd+d^{2}}{-a_{2}+b+c+2d},$$

(7) $$a_{2}=a_{1}+l\sin\theta ,$$

where c represents the camera height; *a*_1_ and *a*_2_ represent the actual position of the fluorescence; *b* represents the height of the fluorescence relative to the reference plane; *x* and *x*1 represent the difference in the fluorescence in the two mirrors, respectively; *d* represents the distance from the bottom of the mirror to the center in the reference plane; *l* is the distance of the camera’s movement between the two frames; and *θ* is the angle between the mirror and the direction of travel.

All cases follow the rules proposed below:

IF (there are fluorescent blobs in the center area) THEN

Case1:

Choose the blob center as the plant center;

ELSE IF (there are fluorescent blobs in four mirror areas) THEN

Case2:

Connect the TL and BR;

Connect the TR and BL;

Choose the intersection of two lines segments as the plant center;

ELSE IF (there are fluorescent blobs in three mirror areas) THEN

Case3:

Connect the TL and BR;

Make a vertical line of the mirror edge through the TR;

Choose the intersection of two lines segments as the plant center;

Case4:

Connect the TL and BR;

Make a vertical line of the mirror edge through the BL;

Choose the intersection of two lines segments as the plant center;

Case5:

Connect the TR and BL;

Make a vertical line of the mirror edge through the TL;

Choose the intersection of two lines segments as plant center;

Case6:

Connect the TR and BL;

Make a vertical line of the mirror edge through the BR;

Choose the intersection of two lines segments as plant center;

ELSE IF (there are fluorescent blobs in two non-opposite mirror area) THEN

Case7:

Make a vertical line of the mirror edge through the TR;

Make a vertical line of the mirror edge through the BR;

Choose the intersection of two lines segments as the plant center;

Case8:

Make a vertical line of the mirror edge through the TL;

Make a vertical line of the mirror edge through the BL;

Choose the intersection of two lines segments as plant center;

Case9:

Make a vertical line of the mirror edge through the TL;

Make a vertical line of the mirror edge through the TR;

Choose the intersection of two lines segments as plant center;

Case10:

Make a vertical line of the mirror edge through the BL;

Make a vertical line of the mirror edge through the BR;

Choose the intersection of two lines segments as plant center;

ELSE IF (there are fluorescent blob in two opposite mirror areas) THEN

Case11:

Apply the opposite algorithm to BL and TR.

Calculate the plant center;

Case12:

Apply the opposite algorithm to BR and TL.

Calculate the plant center;

ELSE IF (there are fluorescent blobs in one mirror area) THEN

Case13:

Apply the opposite algorithm to TL.

Calculate the plant center;

Case14:

Apply the opposite algorithm to BL.

Calculated the plant center;

Case15:

Apply the opposite algorithm to TR.

Calculate the plant center;

Case16:

Apply the opposite algorithm to BR.

Calculate the plant center;

ELSE

The plant center is not found.

### 2.5. Statical Analysis 

All statistical data were processed by IBM SPSS 27 (SPSS Inc., Chicago, IL, USA). An analysis of variance and Tukey honestly significant difference (HSD) were used to assess the different intensities. The effect of concentration on fluorescence performance and plant growth was first analyzed. After that, the effect of different immersion times on fluorescence performance and crop growth safety was analyzed. The figure was plotted using Origin software (OriginLab Corporation, Northampton, MA, USA).

## 3. Results

### 3.1. Visibility and Distribution of Rh-B Fluorescence in Soybean

**Figure 5:**
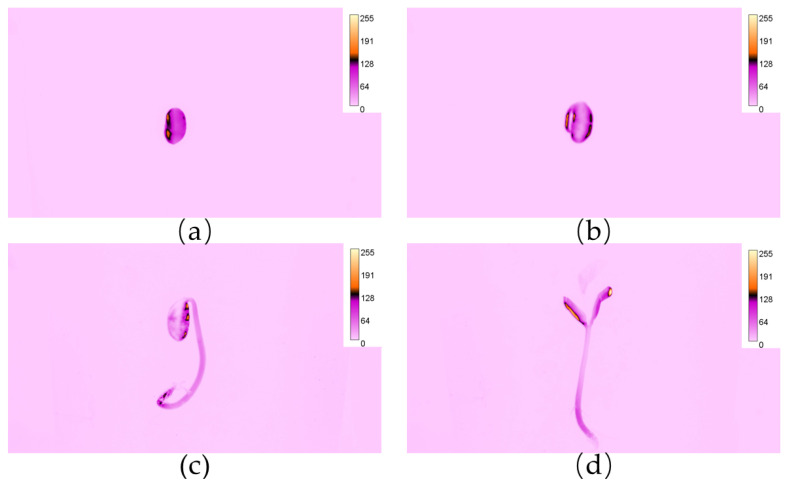
Fluorescence distribution map of soybean plants at different stages: (**a**) seed imbibition, (**b**) germination stage, (**c**) emergence stage, (**d**) cotyledon stage.

A series of soybean fluorescent images were taken during the growth of soybean seedlings to observe the distribution of fluorescence in treated soybean seedlings. Figure 5 shows the four stages of soybean development. A fluorescent image of a soybean seed after it was soaked in a Rh-B solution is shown in Figure 5a. The soybean seeds mainly consisted of a seed coat, cotyledons, hypocotyl, radicle, and embryo. At the right temperature and humidity, seeds will start germinating after absorbing sufficient water. The radicle, which eventually develops into the root of the crop, was the first to grow. The hypocotyl will elongate and push the cotyledons out of the soil after the radicle grows and then develop into the stem. Then, the cotyledons unfold and turn green, which will serve as the main organ for photosynthesis before the embryo grows into the first real leaf. The photos of seeds treated with Rh-B captured by the CMOS camera under a green-exciting light showed high fluorescence intensity. As shown in Figure 5b and Table 1, the fluorescence intensity of the radicle was lower than that of the seeds. As the radicle elongated, its fluorescence decreased. Figure 5c shows the fluorescence distribution of a seedling before its cotyledons unfold. Hypocotyls located closer to the radicle have a higher fluorescence than areas further away from the radicle. Their fluorescence intensity decreased after cotyledon unfolding, compared to the seed period. The fluorescence intensity at the distal end of the cotyledon was higher than that at the proximal end. Figure 5d shows a fluorescent image of a soybean after its first true leaves have grown, which shows that the intensity of the upper part of the cotyledons, including the epicotyl and true leaves, is very low. The fluorescence intensity of each part of the soybean seedlings was positively correlated with their volume share of the native organs in soybean seeds. Therefore, it can be assumed that the fluorescence intensity of each site depends on the quantity of Rh-B contained in that site.

### 3.2. Effects of Different Rh-B Concentrations and Different Soaking Times on the Fluorescence Visibility of Soybean Seedlings

The relationship between the fluorescence intensity within the plant and the concentration of the solution used to soak the seeds was derived from this experiment. In a previous study, 60 and 120 ppm solutions were used to soak soybean seeds, which revealed that the fluorescence intensity of plants treated with 120 ppm Rh-B was significantly higher than that of plants treated with 60 ppm. Therefore, the concentration gradient of the 60–180 ppm groups was subdivided to investigate the relationship between the concentration of Rh-B solutions and the fluorescence of soybean seedlings. Five concentrations of Rh-B (60, 90, 120, 150, 180 ppm) solutions were used to soak soybean seeds to determine the most appropriate treatment. Table 2 and Table 3 showed the fluorescence intensity statistics of soybean plants treated with different Rh-B concentrations. There was a significant positive correlation between the fluorescence intensity of the soybean crop and the concentration of Rh-B solution within the concentration range of 90–180 ppm. The concentration of the Rh-B solutions was expanded to 1440 ppm. Five concentration gradients were selected for soaking soybean seeds for their fluorescence imaging and measurement. Different exposure times were chosen to obtain these fluorescence images because of the different incubation times and concentrations chosen for the two groups of soybeans. In the 90–1440 ppm Rh-B solution-soaked soybean seed tests, the fluorescence intensity of soybean plants under the excitation light tended to rise and then fall as the solution concentration increased. The fluorescence of soybean plants reached a maximum when the Rh-B solution concentration was 360 ppm. Soybeans were immersed in Rh-B solutions for durations of 12, 24, 36, 48, and 60 h, respectively, to investigate the impact of soaking time on soybean fluorescence intensity. Table 4 shows the fluorescence intensity statistics of soybean plants treated with Rh-B concentrations for different lengths of time. The findings indicated that once the soaking duration exceeded 24 h there was no statistically significant effect on soybean fluorescence intensity. However, the fluorescence intensity of soybean plants with a soaking period of 24 h was significantly higher than that of soybean plants with a soaking period of 12 h.

### 3.3. Effects of Rh-B Treatment on the Growth of Soybean Plants

The effects of different concentrations and treatment times on seed germination and biomass (height and weight) were studied. Germination rate is a significant indicator used to test seed quality. As shown in Table 5, the germination rate of soybean seeds soaked in 0–180 ppm Rh-B solutions was recorded to observe the effect of Rh-B concentrations on seed germination. There were no significant differences in germination rate when soybean seeds were immersed in 0–180 ppm Rh-B solutions for 24 h. After measuring their fresh weight, they were dried in a drying oven for 24 h and then removed to measure their dry weight. A one-way analysis of variance (ANOVA) was performed using SPSS software 27 and no significant differences were found. This proved that the concentration of the 0–180 ppm groups had no significant effect on soybean growth. To further explore the effect of soaking concentration on the growth of soybean plants, the plant height and weight of soybeans after soaking their seeds in 90–1440 ppm Rh-B solution are recorded in Table 6 and Table 7, respectively. The results show that when the concentration of Rh-B solution used to soak soybean seeds reached 360 ppm, it had an inhibitory effect on soybean growth. Thus, a treatment concentration of 180 ppm is recommended for proper soybean growth. Subsequently, the germination of soybean seeds which were soaked in 90 ppm Rh-B solutions for 12–60 h was recorded in Table 8. The results showed that there was no significant effect when the soaking time was less than 48 h. When the soaking time exceeded 48 h, the germination rate of the soybeans decreased significantly.

### 3.4. Decay of Visibility of Rh-B Fluorescence in Soybean

**Figure 6:**
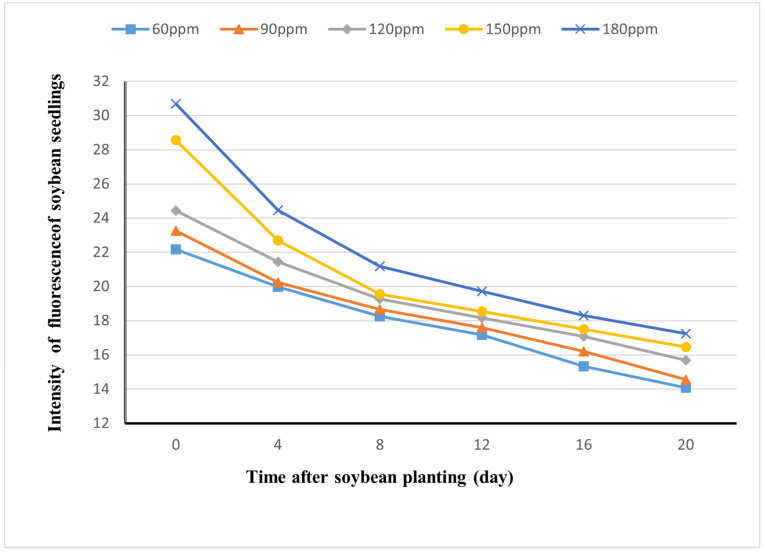
Fluorescence attenuation trend chart of seeds soaked in 60–180 ppm Rh-B.

The fluorescence decay of Rh-B was observed through fluorescence images collected every four days. The soybean seeds, which were soaked in six different concentrations of Rh-B solution, were sown into nutrient soil after their germination. The mean maximum intensity of the control group was 4, so a minimum threshold of 4 was used to filter out the intensity of non-fluorescence. Figure 6 demonstrated the decay of soybean fluorescence with time after different concentrations of immersion. The correlation between fluorescence concentration and time was confirmed at a significance level of 0.01. Over the first four days, the fluorescence decreased the fastest. The decay of fluorescence intensity gradually slowed down with increasing time after sowing. Finally, the fluorescence intensity of the soybeans was still significantly different from the background.

### 3.5. Result of Identification and Location of Soybean Plants

The accuracy of the proposed algorithm was tested using the constructed imaging system. A total of 300 images of different growth periods of soybeans were collected. In the early soybean growth period, the fluorescence on the soybean cotyledons could be captured directly in the central region, in most cases. Based on the algorithm, the position of the soybean could be accurately located. However, in the late soybean growth stage, when the cotyledons were obscured by leaves, four mirrors were utilized to add side views of the fluorescence to help locate the soybeans. As shown in Table 9, the following six cases were included in the collected image set: fluorescence in the center area, four areas, three areas, two non-opposed areas, two opposed areas, and in a single area. Their numbers were 32, 67, 54, 65, 44, and 38, respectively. The algorithm is considered to have successfully located the center of the crop if the actual pixel point of the crop is located within a circle, with the pixel point calculated by the algorithm as the center and 43.5 pixels (0.5 cm) as the radius. The positions of the soybean plants were successfully obtained in all scenarios. The highest localization accuracy was obtained with direct center extraction, followed by the scenario in which concatenation could be performed. The two-sided mirror had a higher localization accuracy than the single-sided mirror. The total accuracy of the detection system was 96.7%.

## 4. Discussion

The aim of this project was to propose a systematic crop signaling-based plant localization technique to automatically detect soybeans. Previous studies have used 60 ppm or 120 ppm Rh-B as a fluorescence trace [40]. Although the designed system successfully detected the stem position of soybeans, the optimal density of Rh-B-treated soybean seeds was not analyzed. In this study, we first investigated the fluorescence performance of Rh-B around 100 ppm. A trend of increasing fluorescence intensity was found with rising concentrations in the range of 60–180 ppm. Because a maximum value of 100 ppm was determined from 1, 10, 100, 1000, and 5000 ppm in Su’s study [33], the fluorescence performance Rh-B concentrations in the range of 90–1440 ppm was examined again. The effects of different immersion times on crop and fluorescence properties were also analyzed. In this study, the concentration and appropriate dose of Rh-B were obtained from the experimental results.

The algorithm proposed in this study was optimized on the basis of UC Davis [40] and Sheng [38]. It further improved the accuracy of crop localization while ensuring fast identification. UC Davis was the first to propose a 3D imaging technique based on crop tags. However, in their study, additional crop labelling work was required. Su resolved this extra work by using a systematic crop signal technique that caused the crop itself to fluoresce [37]. Both algorithms lack further reliable localization techniques in the face of opposing and one-sided mirrors. Therefore, this article optimized this part to make this system better and more reliable. However, this experiment was conducted under laboratory conditions, and its practical feasibility in the field should be further validated in the future. The significant advantage of this technology over traditional image processing technology is that it can still effectively detect soybeans when the weed density is high. Traditional image processing technology and deep learning image processing technology often fail when the weed density is high. Crops cannot be effectively identified due to the serious overlapping of colors, textures, etc. [41,42].

Although the fluorescent labeling method used in this study ultimately detected strong fluorescence only in the cotyledon portion of the soybeans, the survival time of the cotyledons was more than 3 weeks. This period is sufficient to ensure the early field weed control requirements of soybeans, as the most severe period of soybean weed infestation tends to occur early in soybean production. However, the method proposed in this study currently has significant limitations. For instance, it requires additional equipment to provide a dark environment for the fluorescence imaging device. Moreover, while this technology greatly reduces the performance requirements for computers and identification systems, the system is based on the specific fluorescence of crop signals, which directly affects the accuracy of the system. Specifically, the targeted capture of Rh-B fluorescence is achieved through the coordination of bandpass and cutoff filters. Therefore, the precision of these filters will directly impact the system’s accuracy. Additionally, it is undeniable that Rh-B, as a fluorescent dye, inherently possesses some level of toxicity. Although this study has conducted preliminary research on its effects on soybean growth and demonstrated that low doses of Rh-B solution do not impact soybean production, the broader impact of Rh-B on farmland and other organisms throughout the agricultural production process has not been evaluated. For example, fallen leaves may contain incompletely degraded Rh-B, which could pose a hazard to the soil. Hence, further research support is still needed before the practical application of this study’s findings. If this research can be successfully applied, its cost will be lower than other intelligent robots because it does not require high computing power and its maintenance is simpler. In addition, the development of this technology has provided ideas for other crop signal technologies to follow. The means for more robust crop signals will be available in the future to achieve precise inter-plant localization with this identification system. This system is based on a unique fluorescence signal technology that does not require the use of crop texture, color, or other features. The crop’s location can be quickly extracted by the proposed algorithm, which does not rely on expensive computing power, providing a significant cost-saving effect. Since it is based on a unique fluorescence signal, it is easier to migrate to other crops that can be grown using this method without the need for new algorithms and equipment, making it highly generalizable. The ability to capture fluorescence is the key to the implementation of this system. It is necessary to build a dark environment and lighting system in outdoor environments. In future work, the construction of outdoor dark rooms and the optimization of the outdoor algorithm will be focused on.

## 5. Conclusions

In this study, a novel soybean crop detection technique based on the crop signaling technique was proposed. The fluorescence distribution properties of systemic crop compounds in soybean and their effects on soybean plant growth were explored. Hypocotyls located closer to the radicle have a higher fluorescence than areas further away from the radicle. The fluorescence intensity at the distal end of the cotyledon was higher than that at the proximal end. The experimental results showed that the treatment of soybean seeds with 360 ppm Rh-B for 24 h obtained the highest fluorescence intensity, but inhibited crop growth. A treatment with 180 ppm Rh-B for 24 h was the recommended dosage, which resulted in significant fluorescence and did not affect crop growth. The fluorescence produced lasted for 20 days, ensuring a stable signal during the early stages of growth. The proposed algorithm for locating soybean stalk centers based on Rh-B crop signal technology achieved good localization results under static laboratory conditions, with an average recognition accuracy of 96.7%. However, its dynamic recognition effect and field effects have not yet been verified, which will be the focus of future work. This work provides ideas for early intra-row weed control for soybeans in the field. This study demonstrates the feasibility of using fluorescent compounds to mark crop seedlings for crop weed detection and identification and provides an important reference for crop weed detection research.

## Figures and Tables

**Table 1:** The fluorescence intensity of different parts of soybean seedlings.

Area	Radicle	Hypocotyl	Cotyledon	Epicotyl	True Leaf
Intensity	9.781 ± 0.124	11.495 ± 0.132	58.438 ± 2.651	4.974 ± 0.144	3.988 ± 0.234

**Table 2:** The statistics of the average fluorescence intensity of soybean plants according to their seeds being treated with 0–180 ppm Rh-B solutions.

Rh-B Concentration (ppm)	Intensity			ANOVA *p* Value
	Mean ± SD	Max	Min	
0	4.00 ± 0.05	4.04	4.00	*p* < 0.001
60	18.35 ± 3.07	19.26	14.15	*p* > 0.05
90	18.50 ± 3.10	19.87	14.55	
120	19.28 ± 3.00	20.05	14.64	
150	20.27 ± 4.29	21.22	15.36	
180	21.97 ± 4.95	23.54	16.78	

**Table 3:** The statistics of the average fluorescence intensity of soybean plants according to their seeds being treated with 90–1440 ppm Rh-B solutions.

Days after Germination (d)	Rh-B Concentration (ppm)	Mean ± SD	Max	Min	ANOVA
1	90	116.50 ± 12.64	140.04	104.56	*p* < 0.001
	180	164.00 ± 9.95	181.78	153.25	
	360	236.37 ± 13.25	251.18	214.56	
	720	156.62 ± 9.72	171.36	143.27	
	1440	129.75 ± 12.55	151.98	110.56	
5	90	102.75 ± 12.48	124.48	86.58	
	180	156.12 ± 14.99	181.94	132.37	
	360	198.87 ± 15.25	221.66	176.48	
	720	146.37 ± 9.16	160.31	136.95	
	1440	126.75 ± 19.63	168.24	111.34	
10	90	98.87 ± 13.99	124.27	78.56	
	180	148.37 ± 13.08	159.35	124.87	
	360	155.25 ± 16.06	184.41	32.64	
	720	126.25 ± 6.88	135.61	118.47	
	1440	123.37 ± 14.72	151.51	102.14	
15	90	98.87 ± 13.11	115.61	76.25	
	180	125.50 ± 17.44	149.24	102.86	
	360	136.00 ± 11.97	159.36	124.94	
	720	114.12 ± 6.31	122.48	105.64	
	1440	112.37 ± 15.66	140.54	98.38	
20	90	92.37 ± 9.65	105.56	76.34	
	180	111.62 ± 4.83	115.65	104.64	
	360	124.62 ± 20.33	157.61	101.69	
	720	108.25 ± 8.53	116.14	90.82	
	1440	100.12 ± 7.79	109.87	88.46	

**Table 4:** The statistics of the average fluorescence intensity of soybean plants according to how long their seeds were treated with 360 ppm Rh-B solutions (0–60 h).

Soaking Time	Fluorescence Intensity			ANOVA	Tukey HSD
	Mean ± SD	Max	Min	*p* Value	*p* Value
0	4.12 ± 0.23 c	5.12	4.00	*p* < 0.001	*p* < 0.001
12 h	76.40 ± 14.57 b	92.54	65.01		*p* < 0.001
24 h	137.70 ± 25.07 a	159.35	124.36		*p* < 0.001 for 0 h and 12 h, *p* > 0.05 for others
36 h	141.40 ± 25.86 a	196.36	111.47		*p* < 0.001 for 0 h and 12 h, *p* > 0.05 for others
48 h	138.20 ± 16.07 a	203.12	112.64		*p* < 0.001 for 0 h and 12 h, *p* > 0.05 for others
60 h	134.40 ± 61.18 a	158.86	114.76		*p* < 0.001 for 0 h and 12 h, *p* > 0.05 for others

If there is a significant difference between two groups, they will be labeled with different letters. If there is no significant difference between two groups, they will be labeled with the same letter.

**Table 5:** Statistical values of the biomass of soybean plants treated with 0–180 ppm Rh-B solutions.

Concentration (ppm)	Germination Rate	Height (cm)			Weight (g)			ANOVA
		Mean ± SD	Max	Min	Mean ± SD	Max	Min	*p* Value
0 ppm	0.96	24.3 ± 3.3	28.5	22.5	2.863 ± 0.866	3.364	2.363	*p* > 0.05
60 ppm	0.97	25.1 ± 2.1	26.4	19.9	2.364 ± 0.786	2.743	1.985	
90 ppm	0.95	25.7 ± 2.2	28.2	24.1	2.491 ± 1.002	3.007	1.976	
120 ppm	0.95	25.1 ± 6.5	28.4	23.5	2.857 ± 0.906	3.308	2.407	
150 ppm	0.97	25.2 ± 2.6	30.3	24.2	2.269 ± 0.770	2.620	1.918	
180 ppm	0.95	24.6 ± 2.9	28.4	23.4	2.348 ± 0.911	2.802	1.896	

**Table 6:** Statistical values of the height of soybean plants treated with 90–1440 ppm Rh-B solutions.

Rh-B Concentration (ppm)	Germination Rate	Height (cm)			Tukey HSD
		Mean ± SD	Max	Min	*p* Value
0 ppm	0.96	36.4 ± 5.2 a	44.3	25.2	*p* > 0.05 for 90 ppm and 180 ppm, *p* < 0.05 for others
90 ppm	0.97	37.2 ± 7.3 a	45.2	24.3	0.176 for 180 ppm, 0.524 for 360 ppm, 0.018 for 720 ppm, 0.003 for 1440 ppm
180 ppm	0.95	35.2 ± 6.2 ab	43.2	24.8	0.524 for 90 ppm, 0.521 for 360 ppm, 0.117 for 720 ppm, 0.023 for 1440 ppm
360 ppm	0.95	33.1 ± 4.6 ab	44.5	22.6	0.176 for 90 ppm, 0.521 for 180 ppm, 0.330 for 720 ppm, 0.066 for 1440 ppm
720 ppm	0.97	30.4 ± 5.3 b	39.0	22.6	0.018 for 90 ppm, 0.117 for 180 ppm, 0.330 for 360 ppm, 0.269 for 1440 ppm
1440 ppm	0.95	26.9 ± 7.8 b	37.9	17.5	0.003 for 90 ppm, 0.023 for 180 ppm, 0.066 for 360 ppm, 0.269 for 720 ppm

If there is a significant difference between two groups, they will be labeled with different letters. If there is no significant difference between two groups, they will be labeled with the same letter.

**Table 7:** Statistical values of the weight of soybean plants treated with 90–1440 ppm Rh-B solutions.

Rh-B Concentration (ppm)	Weight (g)			Tukey HSD
	Mean ± SD	Max	Min	*p* Value
0 ppm	4.665 ± 1.236 ab	6.187	3.188	0.377 for 90 ppm, 0.987 for 180 ppm, 0.186 for 360 ppm, *p* < 0.05 for 720 ppm and 1440 ppm
90 ppm	5.253 ± 1.357 ab	7.750	3.403	0.377 for 0 ppm, 0.386 for 180 ppm, *p* < 0.05 for 360 ppm, 720 ppm, 1440 ppm
180 ppm	4.677 ± 1.503 ab	6.401	1.938	0.987 for 0 ppm, 0.386 for 90 ppm, 0.181 for 360 ppm, *p* < 0.05 for 720 ppm, 1440 ppm
360 ppm	3.754 ± 0997 b	5.225	2.350	0.186 for 0 ppm, 0.181 for 180 ppm, 0.379 for 720 ppm, 0.328 for 1440 ppm
720 ppm	3.216 ± 1.404 b	6.306	1.482	0.379 for 360 ppm, 0.770 for 1440 ppm, *p* < 0.05 for 0 ppm, 90 ppm, 180 ppm
1440 ppm	3.017 ± 0.525 b	3.546	2.350	0.328 for 360 ppm, 0.770 for 1440 ppm, *p* < 0.05 for 0 ppm, 90 ppm, 180 ppm

If there is a significant difference between two groups, they will be labeled with different letters. If there is no significant difference between two groups, they will be labeled with the same letter.

**Table 8:** Statistical values of the biomass of soybean plants treated with 90 ppm Rh-B solutions for 0–60 h.

Days after Germination (d)	Germination Rate	Height (cm)			ANOVA	Tukey HSD
		Mean ± SD	Max	Min		*p* Value
0	0.97	25.2 ± 1.6 a	27.6	22.8	*p* < 0.001	*p* < 0.001 for 60 h, *p* > 0.05 for others
12 h	0.96	25.4 ± 1.9 a	28.9	22.1		*p* < 0.001 for 60 h, *p* > 0.05 for others
24 h	0.97	25.1 ± 3.1 a	29.3	19.8		*p* < 0.001 for 60 h, *p* > 0.05 for others
36 h	0.95	23.2 ± 2.7 ab	28.9	20.3		*p* > 0.05
48 h	0.80	22.1 ± 4.1 ab	27.6	14.8		*p* > 0.05
60 h	0.63	19.6 ± 2.3 b	24.6	16.9		*p* < 0.01 for 0 h, 12 h, 24 h, *p* > 0.05 for others

If there is a significant difference between two groups, they will be labeled with different letters. If there is no significant difference between two groups, they will be labeled with the same letter.

**Table 9:** Results of multi-view soybean localization algorithm in different occlusion scenarios.

Cases	Binary Image	Positioning Image	Amount	Accuracy
Central area	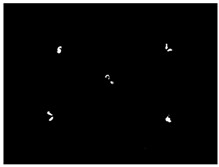	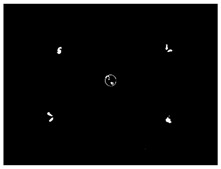	32	100%
Four areas	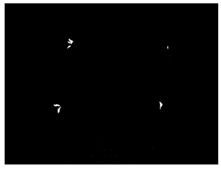	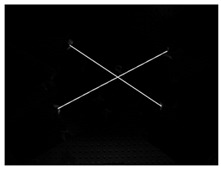	67	100%
Three areas	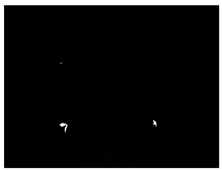	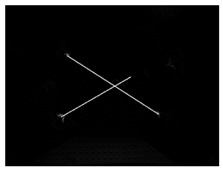	54	100%
Non-opposite two areas	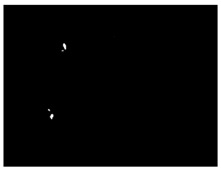	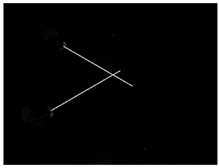	65	96.9%
Opposite areas	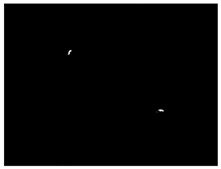	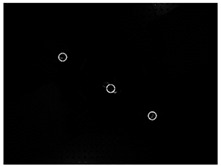	44	93.18%
One area	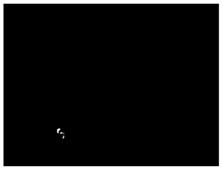	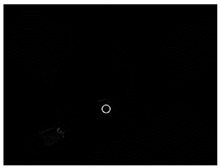	38	86.8%

## Data Availability

Data are available on request due to privacy.

## References

[1] Ferreir A.D.S., Freitas D.M., da Silva G.G., Pistori H., Folhes M.T. (2017). Weed detection in soybean crops using ConvNets. Comput. Electron. Agric..

[2] Ahmed S., Kumar V., Alam M., Dewan M.R., Bhuiyan K.A., Miajy A.A., Saha A., Singh S., Timsina J., Krupnik T.J. (2021). Integrated weed management in transplanted rice: Options for addressing labor constraints and improving farmers’ income in Bangladesh. Weed Technol..

[3] Bakhshipour A., Jafari A., Nassiri S.M., Zare D. (2017). Weed segmentation using texture features extracted from wavelet sub-images. Biosyst. Eng..

[4] Chang C.-L., Xie B.-X., Chung S.-C. (2021). Mechanical control with a deep learning method for precise weeding on a farm. Agriculture.

[5] Dai X., Xu Y., Zheng J., Song H. (2019). Analysis of the variability of pesticide concentration downstream of inline mixers for direct nozzle injection systems. Biosyst. Eng..

[6] Fan X., Chai X., Zhou J., Sun T. (2023). Deep learning based weed detection and target spraying robot system at seedling stage of cotton field. Comput. Electron. Agric..

[7] Fennimore S.A., Slaughter D.C., Siemens M.C., Leon R.G., Saber M.N. (2016). Technology for automation of weed control in specialty crops. Weed Technol..

[8] Gaur M., Faldu K., Sheth A. (2021). Semantics of the black-box: Can knowledge graphs help make deep learning systems more interpretable and explainable?. IEEE Internet Comput..

[9] Grassini P., La Menza N.C., Edreira J.I.R., Monzón J.P., Tenorio F.A., Specht J.E. (2021). Soybean. Crop Physiology Case Histories for Major Crops.

[10] Hasan A.M., Sohel F., Diepeveen D., Laga H., Jones M.G. (2021). A survey of deep learning techniques for weed detection from images. Comput. Electron. Agric..

[11] He D., Qiao Y., Li P., Gao Z., Li H., Tang J. (2013). Weed recognition based on SVM-DS multi-feature fusion. Nongye Jixie Xuebao = Trans. Chin. Soc. Agric. Mach..

[12] Jugulam M., Shyam C. (2019). Non-target-site resistance to herbicides: Recent developments. Plants.

[13] Li B., Wang G., Fan C., Su L., Xu X. (2009). Effects of different tillage methods on weed emergence in summer soybean field. J. Hebei Agric. Sci.

[14] Li Y., Guo Z., Shuang F., Zhang M., Li X. (2022). Key technologies of machine vision for weeding robots: A review and benchmark. Comput. Electron. Agric..

[15] Liu B., Bruch R. (2020). Weed detection for selective spraying: A review. Curr. Robot. Rep..

[16] Liu K., Liu K. (1997). Chemistry and nutritional value of soybean components. Soybeans: Chemistry, Technology, and Utilization.

[17] Mattivi P., Pappalardo S.E., Nikolić N., Mandolesi L., Persichetti A., De Marchi M., Masin R. (2021). Can commercial low-cost drones and open-source GIS technologies be suitable for semi-automatic weed mapping for smart farming? A case study in NE Italy. Remote Sens..

[18] Melander B., Rasmussen G. (2001). Effects of cultural methods and physical weed control on intrarow weed numbers, manual weeding and marketable yield in direct-sown leek and bulb onion. Weed Res..

[19] Nguyen T.T., Slaughter D.C., Fennimore S.A., Vuong V.L. (2017). Designing and evaluating the use of crop signaling markers for fully automated and robust weed control technology. Proceedings of the 2017 ASABE Annual International Meeting.

[20] Onyango C.M., Marchant J. (2003). Segmentation of row crop plants from weeds using colour and morphology. Comput. Electron. Agric..

[21] Rai N., Zhang Y., Villamil M., Howatt K., Ostlie M., Sun X. (2024). Agricultural weed identification in images and videos by integrating optimized deep learning architecture on an edge computing technology. Comput. Electron. Agric..

[22] Raja R., Nguyen T.T., Slaughter D.C., Fennimore S.A. (2020). Real-time robotic weed knife control system for tomato and lettuce based on geometric appearance of plant labels. Biosyst. Eng..

[23] Raja R., Nguyen T.T., Slaughter D.C., Fennimore S.A. (2020). Real-time weed-crop classification and localisation technique for robotic weed control in lettuce. Biosyst. Eng..

[24] Raja R., Nguyen T.T., Vuong V.L., Slaughter D.C., Fennimore S.A. (2020). RTD-SEPs: Real-time detection of stem emerging points and classification of crop-weed for robotic weed control in producing tomato. Biosyst. Eng..

[25] Raja R., Slaughter D.C., Fennimore S.A., Nguyen T.T., Vuong V.L., Sinha N., Tourte L., Smith R.F., Siemens M.C. (2019). Crop signalling: A novel crop recognition technique for robotic weed control. Biosyst. Eng..

[26] Raja R., Slaughter D.C., Fennimore S.A., Siemens M.C. (2023). Real-time control of high-resolution micro-jet sprayer integrated with machine vision for precision weed control. Biosyst. Eng..

[27] Reedha R., Dericquebourg E., Canals R., Hafiane A. (2022). Transformer neural network for weed and crop classification of high resolution UAV images. Remote Sens..

[28] Ronchi C., Silva A., Korres N., Burgos N., Duke S. (2018). Weed Control: Sustainability, Hazards and Risks in Cropping Systems Worldwide.

[29] Ruigrok T., van Henten E.J., Kootstra G. (2023). Improved generalization of a plant-detection model for precision weed control. Comput. Electron. Agric..

[30] Su W.-H. (2020). Crop plant signaling for real-time plant identification in smart farm: A systematic review and new concept in artificial intelligence for automated weed control. Artif. Intell. Agric..

[31] Su W.-H., Fennimore S.A., Slaughter D.C. (2019). Fluorescence imaging for rapid monitoring of translocation behaviour of systemic markers in snap beans for automated crop/weed discrimination. Biosyst. Eng..

[32] Su W.-H., Fennimore S.A., Slaughter D.C. (2020). Development of a systemic crop signalling system for automated real-time plant care in vegetable crops. Biosyst. Eng..

[33] Su W.-H., Sheng J., Huang Q.-Y. (2022). Development of a Three-Dimensional Plant Localization Technique for Automatic Differentiation of Soybean from Intra-Row Weeds. Agriculture.

[34] Su W.-H., Slaughter D.C., Fennimore S.A. (2020). Non-destructive evaluation of photostability of crop signaling compounds and dose effects on celery vigor for precision plant identification using computer vision. Comput. Electron. Agric..

[35] Su W.-H., Zhang J., Yang C., Page R., Szinyei T., Hirsch C.D., Steffenson B.J. (2020). Automatic evaluation of wheat resistance to fusarium head blight using dual mask-RCNN deep learning frameworks in computer vision. Remote Sens..

[36] Villette S., Maillot T., Guillemin J.-P., Douzals J.-P. (2022). Assessment of nozzle control strategies in weed spot spraying to reduce herbicide use and avoid under-or over-application. Biosyst. Eng..

[37] Wang A., Zhang W., Wei X. (2019). A review on weed detection using ground-based machine vision and image processing techniques. Comput. Electron. Agric..

[38] Wang Q., Cheng M., Huang S., Cai Z., Zhang J., Yuan H. (2022). A deep learning approach incorporating YOLO v5 and attention mechanisms for field real-time detection of the invasive weed Solanum rostratum Dunal seedlings. Comput. Electron. Agric..

[39] Wang X., Pan T., Qu J., Sun Y., Miao L., Zhao Z., Li Y., Zhang Z., Zhao H., Hu Z. (2023). Diagnosis of soybean bacterial blight progress stage based on deep learning in the context of data-deficient. Comput. Electron. Agric..

[40] Zhang W. (2021). The Identification Technology Research of Corn Seedlings and Weeds based on Machine Vision to Target Application System. Master’s Thesis.

[41] Zhang X., Xie Z., Zhang N., Cao C. (2012). Weed recognition from pea seedling images and variable spraying control system. Nongye Jixie Xuebao = Trans. Chin. Soc. Agric. Mach..

[42] Zhang Y., Staab E.S., Slaughter D.C., Giles D.K., Downey D. (2012). Automated weed control in organic row crops using hyperspectral species identification and thermal micro-dosing. Crop Prot..

